# Delayed radial nerve palsy caused by elbow heterotopic ossification following a Monteggia fracture

**DOI:** 10.12701/jyms.2026.43.43

**Published:** 2026-06-25

**Authors:** Bum-Jin Shim

**Affiliations:** Department of Orthopedic Surgery, Yeungnam University Hospital, Yeungnam University College of Medicine, Daegu, Korea

After falling from a height of 3 m, a 60-year-old man sustained multiple injuries, including rib, blowout, and skull fractures. Orthopedically, he presented with a radial head dislocation and an ulnar shaft fracture (i.e., a Monteggia fracture) (Fig. 1A). He underwent surgical fixation for the ulnar shaft fracture at another hospital, and 3 weeks later, due to worsening hemopneumothorax, he was transferred to our hospital for specialized treatment in the cardiothoracic surgery intensive care unit.

He complained of wrist drop, inability to extend his fingers, numbness over the dorsoradial aspect of the hand, and limited elbow motion. The flexion/extension of the elbow was 80°/30° compared with 130°/0° on the contralateral side. According to the patient, these symptoms did not present immediately after surgery. Three weeks postoperatively, plain radiography demonstrated heterotopic ossification (HO) around the elbow joint (Fig. 1B). Computed tomography clearly delineated the lesion, which was predominantly located lateral to the radial head (Fig. 1C, 1D). Magnetic resonance imaging demonstrated the radial nerve immediately adjacent to the HO, with evidence of compression (Fig. 2A). Electromyography confirmed radial nerve palsy. Surgical exploration revealed radial nerve compression by the HO. Neurolysis and excision of the ectopic bone were performed (Fig. 2B, 2C). At the 6-month follow-up, elbow motion and neurological symptoms had substantially improved.

HO showed ectopic bone formation in the periarticular soft tissues, with various causes, including trauma or brain injury. Although the prevalence of HO varies depending on the underlying clinical circumstances, it is uncommon [1]. If HO occurs around the elbow joint, the joint’s range of motion can be severely restricted, placing significant limitations on daily life. In addition, although rare, delayed nerve dysfunction can occur because of elbow HO. According to previous literature, the ulnar nerve is most frequently affected; however, involvement of the median or radial nerve has also been reported [2]. The patient developed radial nerve palsy secondary to elbow HO, presenting with wrist drop, finger and thumb extension weakness, and sensory disturbances in the dorsoradial aspect of the hand [3].

In the present case, HO resulted in delayed radial nerve compression. Although uncommon, delayed radial nerve palsy caused by elbow HO should be recognized as a potential complication in patients who present with progressive elbow stiffness, new-onset wrist drop, and finger extension weakness after trauma.

## Figures and Tables

**Fig. 1.**
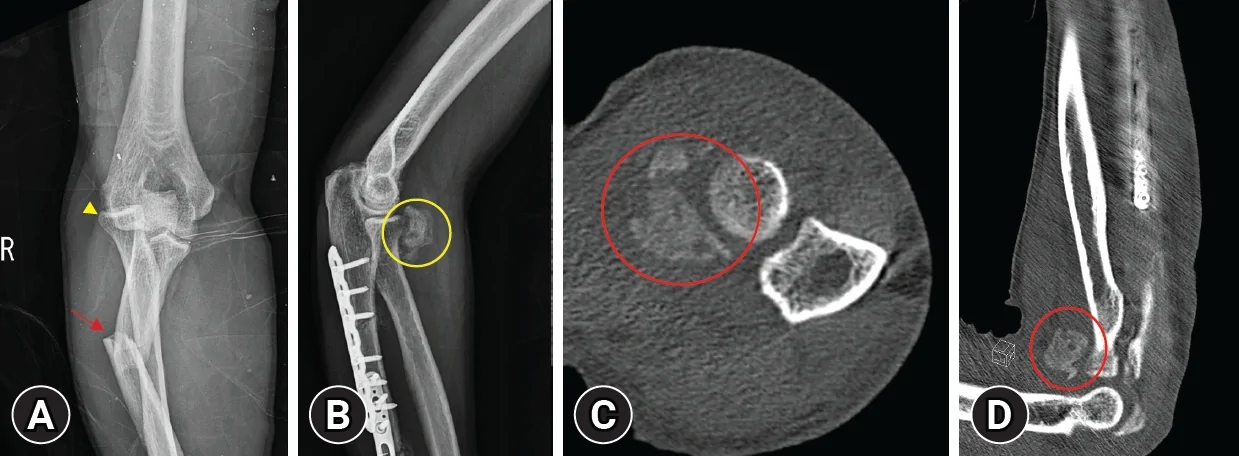
(A) Initial anteroposterior radiographs of the right elbow and forearm demonstrating a Monteggia fracture, consisting of a proximal ulnar shaft fracture (arrow) associated with dislocation of the radial head (arrowhead). (B) Postoperative lateral radiograph of the elbow obtained 3 weeks after fixation, showing heterotopic ossification (yellow circle) adjacent to the radial head and elbow joint. (C) Axial and (D) sagittal computed tomography of the elbow demonstrating heterotopic ossification (red circle) around the elbow joint, predominantly located adjacent to the radial head.

**Fig. 2.**
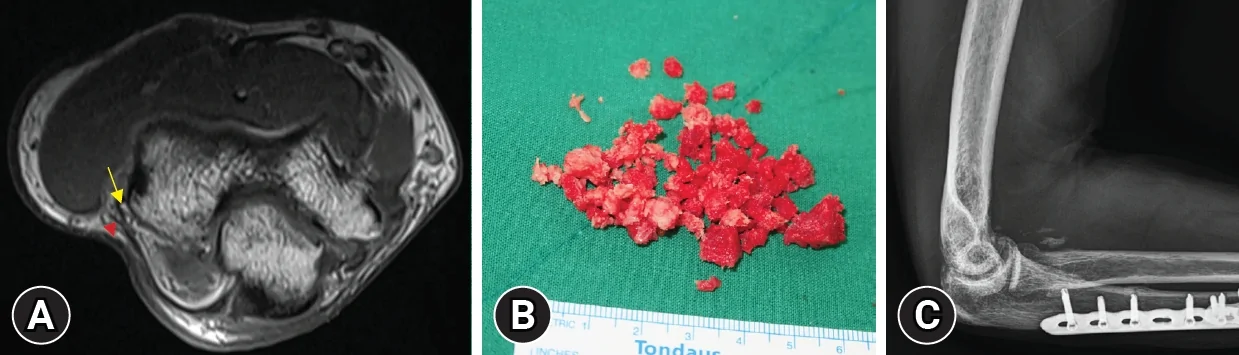
(A) Axial T1-weighted magnetic resonance image demonstrating heterotopic ossification (arrow) causing compression of the radial nerve (arrowhead), corresponding to the patient's radial nerve palsy. (B) Intraoperative photograph showing the excised heterotopic bone fragments following radial nerve exploration, neurolysis, and heterotopic ossification removal. (C) Postoperative lateral radiograph of the elbow demonstrating resection of the heterotopic ossification.
